# Dose- and Time-Dependent Effect of Dietary Blueberries on Diabetic Vasculature Is Correlated with Gut Microbial Signature

**DOI:** 10.3390/antiox12081527

**Published:** 2023-07-30

**Authors:** Adhini Kuppuswamy Satheesh Babu, Chrissa Petersen, Henry A. Paz, Kai Benedict, Miley Nguyen, Madison Putich, Miguel Saldivar-Gonzalez, Ying Zhong, Sydney Larsen, Umesh D. Wankhade, Pon Velayutham Anandh Babu

**Affiliations:** 1 Department of Nutrition and Integrative Physiology, College of Health, University of Utah, Salt Lake City, UT 84112, USA; u6024207@utah.edu (A.K.S.B.); chrissa.petersen@utah.edu (C.P.); kai.benedict@utah.edu (K.B.); miley.nguyen@utah.edu (M.N.); madison.putich@utah.edu (M.P.); u1117617@utah.edu (M.S.-G.); slarsen@union.utah.edu (S.L.); 2 Arkansas Children’s Nutrition Center, Little Rock, Arkansas, AR 72205, USA; hapazmanzano@uams.edu (H.A.P.); zhongying@uams.edu (Y.Z.); uwankhade@uams.edu (U.D.W.); 3 Department of Pediatrics, University of Arkansas for Medical Sciences, Little Rock, AR 72205, USA

**Keywords:** blueberries, dose/time-dependent effect, vascular inflammation, cardiovascular, gut microbes, correlation

## Abstract

Evidence from our lab and others indicates the vascular effects of dietary blueberries. In the present study, we determined dietary blueberries’ dose- and time-dependent effects on diabetic vasculature and their association with gut microbes. Seven-week-old *db*/*db* diabetic male mice were fed a diet supplemented with ± freeze-dried wild blueberry powder (FD-BB) for 4, 8, or 12 weeks (three cohorts). Diets contained 0%, 1.23%, 2.46%, and 3.7% of FD-BB, equivalent to 0, ½, 1, and 1.5 human servings of wild blueberries, respectively. The non-diabetic *db*/*+* mice fed a standard diet served as controls. Metabolic parameters, vascular inflammation, and gut microbiome were assessed. Dietary supplementation of 3.7% FD-BB improved vascular inflammation in diabetic mice without improving systemic milieu in all three cohorts. Blueberries improved diabetes-induced gut dysbiosis depending on blueberry dosage and treatment duration. Spearman’s correlation indicated that the opportunistic microbes and commensal microbes were positively and negatively associated with indices of vascular inflammation, respectively. Dietary blueberries reduced the opportunistic microbe that was positively associated with vascular inflammation (*Desulfovibrio*), and increased the commensal microbe that was negatively associated with vascular inflammation (*Akkermansia*). Dietary blueberries could be a potential adjunct strategy to beneficially modulate gut microbes and improve vascular complications in diabetes.

## 1. Introduction

Diabetes has a major impact on cardiovascular diseases such as atherosclerosis. Cardiovascular disease has been associated with a 2- to 4-fold increase in the mortality rate in individuals with diabetes [1]. In diabetes, high-glucose- and dyslipidemia-induced monocyte binding to the vascular endothelium is the initial step in developing atherosclerosis [2]. The bound monocytes are then transmigrated into the subendothelial space and differentiated into macrophages by the uptake of oxidized LDL [2]. These macrophages are further transformed into lipid-laden foam cells, resulting in vascular inflammation and dysfunction, leading to atherosclerosis [2]. Hence, reducing monocyte binding to the vascular endothelium is a potential target to prevent diabetes-induced vascular disease. Diet plays a major role in enhancing or suppressing atherosclerosis by modulating vascular inflammation [3]. It is well established that diets rich in fruits and vegetables prevent the development of vascular diseases [3]. Blueberries are rich in flavonoids such as anthocyanins and contain more than 18 anthocyanins [2]. Anthocyanins are glycosides of anthocyanidins (cyanidin, delphinidin, malvidin, and peonidin) and carbohydrate components (glucose, galactose, arabinose, etc.) [4]. Evidence from epidemiological, clinical, and preclinical studies indicates the cardiovascular beneficial effects of consuming blueberries [2]. Human studies showed that blueberry intake improves endothelial dysfunction in individuals with metabolic syndrome, increases endothelium-dependent vasodilation in healthy humans, and reduces blood pressure in postmenopausal women [5,6,7].

Gut microbes are essential to host physiology as they produce vitamins, regulate metabolism, modulate the immune system, and metabolize dietary compounds [8,9]. Studies suggest that a balanced healthy gut microbiome prevents many chronic diseases, including cardiovascular disease, diabetes, and colon cancer [10,11]. The host digestive enzymes are inefficient in metabolizing many phytonutrients, and most phytonutrients (such as anthocyanins in blueberries) reach the colon [4]. Gut microbes using microbial enzymes metabolize anthocyanins into small metabolites (such as phenolic acids), which enter the circulation and mediate the biological activities of the dietary blueberries [4]. In addition, a two-way relationship exists between dietary blueberries and gut microbes. Gut microbes metabolize the bioactive components of blueberries, and anthocyanins act as prebiotics through their selective use by commensal microbes to improve the host’s health and reduce the risk of disease [2]. Indeed, gut microbes are essential to mediate the biological activities of the phytonutrients and maintain the host’s health. Conversely, an imbalanced gut microbial population (gut dysbiosis) can adversely affect the host [12].

We recently showed that dietary blueberries at nutritional dosage suppress vascular inflammation and improve vascular dysfunction in diabetic mice, possibly through NOX4-mediated mechanisms [2]. Our in vitro and ex vivo studies further showed that at physiologically relevant dosages, blueberry metabolites ameliorate lipotoxicity-induced vascular dysfunction and suppress endothelial inflammation in the aortic endothelial cells isolated from individuals with type 2 diabetes. In the present study, we investigated the dose- and time-dependent effects of dietary blueberries on diabetic vasculature and diabetes-induced dysbiosis that are largely unknown. We further identified the role of gut microbes in mediating the vascular effects of dietary blueberries by determining the association between the indices of vascular inflammation and gut microbes.

## 2. Materials and Methods

### 2.1. Materials

Dulbecco’s modified Eagle’s medium (DMEM), fetal bovine serum (FBS), and fluorescent dye calcein-AM were purchased from Invitrogen (Carlsbad, CA, USA). DNeasy PowerSoil kit, RNAlater, RNeasy Plus mini kit, QIAzol reagent, QuantiTect reverse transcription kit, qPCR SYBR green master mix, and QuantiTect primers (GAPDH, monocyte chemotactic protein-1 (MCP1), interleukin 8 (IL8), vascular cell adhesion molecule-1 (VCAM1), intercellular adhesion molecule-1 (ICAM1), and E-Selectin) were from Qiagen (Valencia, CA, USA). Protein assay kits were from Bio-Rad (Hercules, CA, USA), and phosphatase and protease inhibitor cocktails were from Sigma-Aldrich (St. Louis, MO, USA). WEHI 78/24 murine monocytic cells were generously provided by Dr. Judith A. Berliner (University of California, Los Angeles, CA, USA).

### 2.2. Experimental Animals

Diabetic *db*/*db* mice with C57BLKS/J background (*db*/*db*; B6.Cg-m^*+*/*+*^Lepr^*db*^) are an established model to study diabetes-induced vascular complications [2,13]. Six-week-old male diabetic (*db*/*db*) mice and control (*db*/*+*) mice were obtained from the Jackson Laboratories (Bar Harbor, ME, USA), and the stock number was 000642. Mice were housed at the University of Utah animal facility (5 mice/cage) under humane conditions and acclimated for a week before the experiments. The mice were kept in controlled artificial lighting conditions following a 12 h light and dark cycle. The temperature was maintained at 23 ± 1 °C and the humidity level was set at 45 ± 5%. The Institutional Animal Care and Use Committee at the University of Utah approved the study protocol 18-09003. All procedures were conducted in accordance with the guidelines outlined in the “Guide for the Care and Use of Laboratory Animals” published by the US National Institute of Health.

### 2.3. Standard-Diet- and Blueberry-Supplemented Diets

*Vaccinium angustifolium* is a species of blueberry commonly known as wild lowbush blueberry. The *Vaccinium* genus belongs to the *Ericaceae* family and wild blueberries (*Vaccinium angustifolium*) were used in the present study. The Wild Blueberry Association of North America (Momence, IL, USA) provided the freeze-dried wild blueberry powder. Standard and blueberry-supplemented diets were purchased from Research Diets Inc. (New Brunswick, NJ, USA). This study aimed to identify the time- and dose-dependent effects of dietary blueberries. Hence, blueberry-supplemented diets were prepared with three different freeze-dried blueberry (FD-BB) powder dosages. Diets contained 1.23%, 2.46%, and 3.7% of FD-BBs (*w*/*w*), equivalent to the human consumption of ~80 g, 160 g, and 240 g of fresh blueberries per day (Table 1). The dosage of FD-BB powder used in this study was determined based on average human consumption patterns (dosages equivalent to ½, 1, and 1.5 human servings of wild blueberries). All the diets were matched for sugar and fiber content. The dosage was calculated based on the Food and Drug Administration (FDA) recommendation for extrapolating the doses from humans to animals by normalizing for body surface area.

### 2.4. Experimental Design

Seven-week-old *db*/*db* male mice were randomly divided into appropriate experimental groups. This study aimed to determine the dose- and time-dependent effects of dietary blueberries. Hence, the mice were initially grouped into three cohorts based on the duration of the treatment (4, 8, or 12 weeks). Each time point (cohorts) consisted of 5 groups (10 mice/group) as follows: (i) Control *db*/*+* mice receiving standard diet (C), (ii) *db*/*db* mice receiving standard diet (D), (iii) *db*/*db* mice receiving 1.23% FD-BB-supplemented diet (DB1), (iv) *db*/*db* mice receiving 2.46% FD-BB-supplemented diet (DB2), and (v) *db*/*db* mice receiving 3.7% FD-BB-supplemented diet (DB3) (Table 1). Mice were treated for 4 weeks (represented by C4, D4, D4B1, D4B2, and D4B3), 8 weeks (represented by C8, D8, D8B1, D8B2, and D8B3), or 12 weeks (represented by C12, D12, D12B1, D12B2, and D12B3), as shown in Table 1.

### 2.5. Measurement of Metabolic Variables and Collection of Tissue Samples

Blood glucose, glucose tolerance, insulin tolerance, and body composition were measured at three different time points (after 4, 8, or 12 weeks of treatment). Food intake and body weight were recorded weekly throughout the study. Contour Next One monitoring system (Bayer, Parsippany, NJ, USA) was used to measure fasting and non-fasting blood glucose concentrations in blood collected from tail veins. Glucose and insulin tolerance tests were performed as we previously described [2,13,14]. To perform the intraperitoneal glucose tolerance test (IPGTT), the mice were subjected to an overnight fast, and a single bolus of glucose (2 g/kg body weight) was administered intraperitoneally. Blood glucose concentrations were measured at 0, 15, 30, 60, and 120 min following glucose administration. To perform the intraperitoneal insulin tolerance test (IPGTT), the mice were subjected to a 4 h fast, and insulin was administered intraperitoneally (0.75 U/kg body weight). Blood glucose concentrations were measured at 0, 15, 30, 60, and 120 min following glucose administration. After treatment (4, 8, or 12 weeks), mice were anesthetized using 2–5% isoflurane, and blood samples were collected via cardiac puncture. The organs and cecum contents were collected from the experimental animals, flash-frozen in liquid nitrogen, and stored at −80 °C. The aortic vessels were dissected free from adherent tissues and used to measure vascular inflammation by assessing monocyte binding to the aortic endothelium and inflammatory molecules. Aorta segments were placed in RNAlater for stabilization of RNA and stored at −80 °C for later PCR analysis. The cecum contents were used for microbial profiling.

### 2.6. Measurement of Vascular Inflammation

The effect of blueberry supplementation on vascular inflammation in diabetic mice was assessed by monocyte binding to the aorta and the expression of inflammatory markers in the aortic vessel we have described with a few modifications [2,13,14]. Briefly, the segments of the abdominal aortae proximal to the iliac bifurcation were used to measure monocyte binding to the vasculature. The aorta was opened to expose the luminal endothelial layer and covered with EBM medium containing 1% heat-inactivated FBS for 10 min at 37 °C. Then, the medium was gently removed, and the exposed endothelium layer of the aorta was incubated with Calcein-AM-labeled mouse monocytic WEHI78/24 cells for 30 min. The unbound monocytes were removed from the aorta by gentle washing. Confocal microscopy was used to visualize and count the bound monocytes. Five images of five frames from each aorta were captured using an Olympus IX73 fluorescence microscope (Olympus, Tokyo, Japan). The vascular inflammation was also measured by determining the expression of inflammatory chemokines (MCP-1 and IL-8) and adhesion molecules (ICAM1, VCAM1, and E-Selectin) by qPCR using SYBR green as we described previously [2,13,14]. Briefly, total RNA was extracted from the aorta using RNeasy Plus mini kit, cDNA was synthesized using an RT-PCR kit, and the expression of inflammatory molecules was measured by qPCR using specific primers and SYBR green. The expression of the housekeeping gene GAPDH was used to normalize the expression.

### 2.7. Microbial Profiling Using 16S rRNA Amplicon Sequencing

Microbial community profiling was carried out using the 16 s rRNA amplification method as we described previously [14]. Bacterial DNA was extracted from the cecum contents using DNeasy PowerSoil Kit, and 50 ng of bacterial DNA was used to amplify the V4 variable region of the 16 S rRNA gene using 515F/806R primers. The primers (forward and reverse) were barcoded to achieve the multiplexing of up to 384 samples per run [15]. The pooled amplicons were then subjected to paired-end sequencing on the Illumina Miseq platform, utilizing a sequencing configuration of 2 × 250 bp. Approximately 30% of PhiX DNA was included in the sequencing run to improve sequencing quality. Raw sequences are available at the NCBI Sequence Read Archive (SRA) under accession no. PRJNA996159.

### 2.8. Bioinformatics Analysis

Miseq Reporter on the instrument computer was used to automate demultiplexing, adapter trimming, and generating fastq files. QIIME 2 platform was used for the subsequent bioinformatics analysis [16]. Denoising was performed through initial quality filtering and applying the Deblur algorithm [17,18]. The representative amplicon sequence variants (ASVs) were utilized to generate the phylogenetic tree with FastTree [19]. To assign the taxonomy, Naives Bayes classifier trained on the Greengenes 13_8 reference was utilized [20]. Rarefaction curves (Supplementary Figure) based on observed ASV were used to evaluate the adequacy of the sampling depth, which was established at 3628-quality-filtered reads per sample. A total of 136 samples passed the established sampling depth and were used in further analyses. α-Diversity metrics (richness, evenness, and diversity) were assessed using the α-Diversity indices such as observed ASV, Shannon Diversity, Evenness, and Dominance. β-Diversity (microbial community differences) was determined using the weighted Unifrac distances, and the principal coordinate analysis (PCoA) plot was used to visualize the results [21].

### 2.9. Statistical Analysis

Prism 8.0 (GraphPad, CA, USA) or SPSS Version 25 (IBM) was used for statistical analyses. Microbiome data were analyzed using the R programming language (version R-4.2.2). One-way ANOVA was employed to analyze metabolic parameters, and Tukey post hoc tests were performed when the main effects were significant. Where appropriate, all data are presented as mean ± standard error of the mean (SEM) and statistical significance was determined at a *p* < 0.05. The relationship between the specific taxa and vascular inflammation was assessed by correlation analysis, as we reported previously [14,22]. Microbiome data (relative abundance of genera) and indices of vascular inflammation (monocyte binding to the aortic vessel and mRNA expression of vascular inflammatory molecules) were used to identify the association between the abundance of specific genera and vascular inflammation through Spearman’s correlation in R [14].

## 3. Results

### 3.1. Dietary Blueberries Did Not Improve Diabetes-Induced Metabolic Alterations

**Figure 1:**
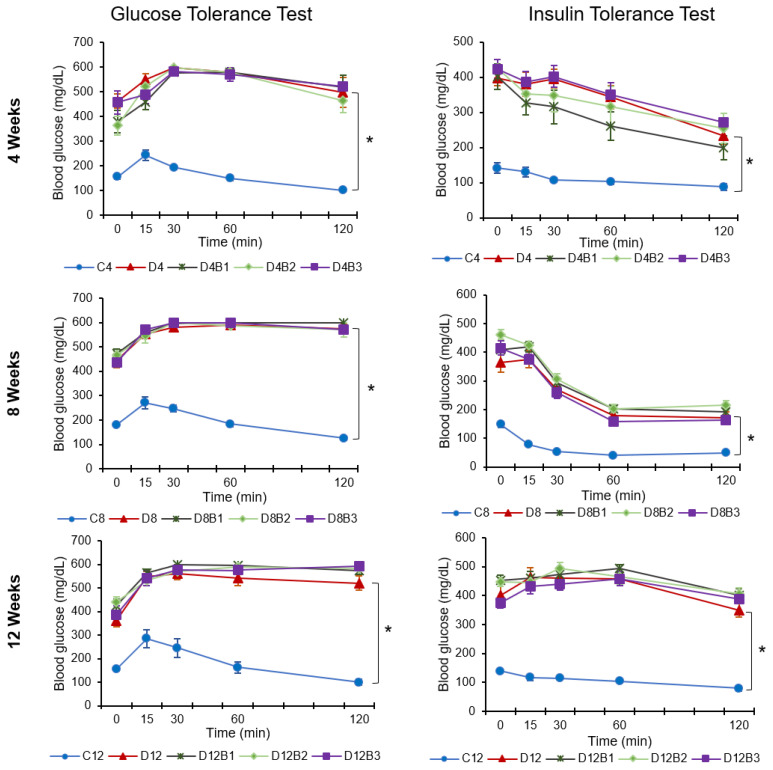
Glucose tolerance test and insulin tolerance test in experimental mice. C4, C8, and C12: non-diabetic (*db*/*+*) mice fed a standard diet for 4, 8, or 12 weeks; D4, D8, and D12: diabetic (*db*/*db*) mice fed a standard diet for 4, 8, or 12 weeks; D4B1, D8B1, and D12B1: diabetic (*db*/*db*) mice fed 1.23% freeze-dried blueberry (FD-BB)-supplemented diet for 4, 8, or 12 weeks; D4B2, D8B2, and D12B2: diabetic (*db*/*db*) mice fed a 2.46% FD-BB-supplemented diet for 4, 8, or 12 weeks; and D4B3, D8B3, and D12B3: diabetic (*db*/*db*) mice fed a 3.7% FD-BB-supplemented diet for 4, 8, or 12 weeks. Values are mean ± SEM (*n* = 8–10). * *p* < 0.05, standard-diet-fed diabetic mice vs. standard-diet-fed control mice.

The effect of dietary blueberries on metabolic parameters (body weight, blood glucose, body fat, lean body mass, and body fluid) was evaluated after 4, 8, and 12 weeks of treatment. The body weight, blood glucose, and body fat were increased, whereas lean body mass was decreased in diabetic (*db*/*db*) mice vs. control (*db*/*+*) mice in all three cohorts (Table 2). However, blueberry supplementation (1.23%, 2.46%, or 3.7% in diet) did not improve these metabolic parameters with 4, 8, or 12 weeks of treatment (Table 2). Further, diabetic mice exhibited impaired glucose and insulin tolerance that was not improved with blueberry supplementation (Figure 1).

### 3.2. Dietary Blueberries Exhibit a Dose-Dependent Ability to Suppress Diabetes-Induced Vascular Inflammation

**Figure 2:**
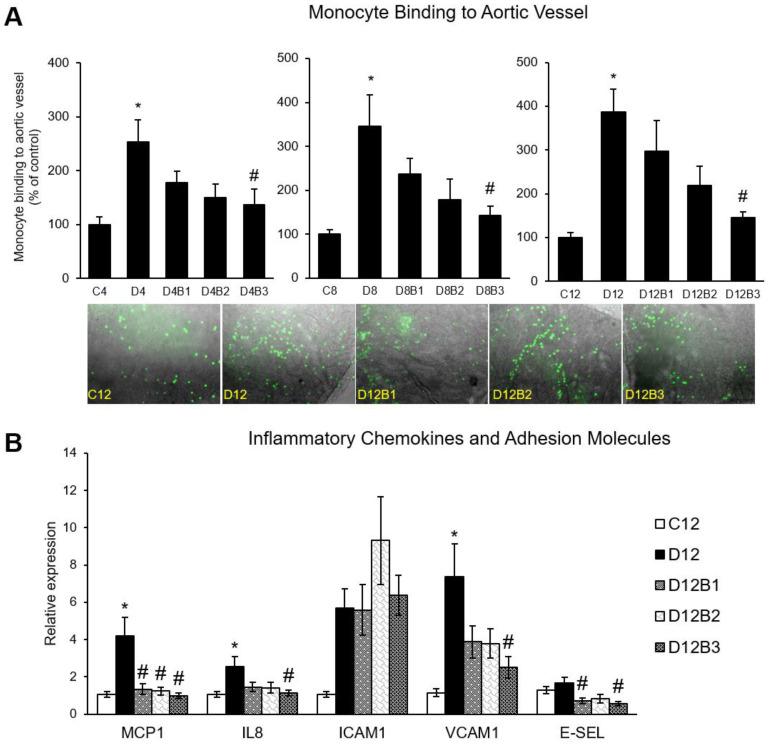
(**A**) Monocyte binding to aortic vessels (4-week, 8-week, and 12-week cohorts), and (**B**) inflammatory chemokines and adhesion molecules (12-week cohort). C4, C8, and C12: non-diabetic (*db*/*+*) mice fed a standard diet for 4, 8, or 12 weeks; D4, D8, and D12: diabetic (*db*/*db*) mice fed a standard diet for 4, 8, or 12 weeks; D4B1, D8B1, and D12B1: diabetic (*db*/*db*) mice fed 1.23% freeze-dried blueberry (FD-BB)-supplemented diet for 4, 8, or 12 weeks; D4B2, D8B2, and D12B2: diabetic (*db*/*db*) mice fed a 2.46% FD-BB-supplemented diet for 4, 8, or 12 weeks; and D4B3, D8B3, and D12B3: diabetic (*db*/*db*) mice fed a 3.7% FD-BB-supplemented diet for 4, 8, or 12 weeks. Values are mean ± SEM (*n* = 6–8). * *p* < 0.05, standard-diet-fed diabetic mice vs. standard-diet-fed control mice; and # *p* < 0.05, blueberry-fed diabetic mice vs. standard-diet-fed diabetic mice.

The dose- and time-dependent effects of dietary blueberry supplementation on endothelial inflammation were assessed by monocyte binding to the aortic vessel. As expected, there was an increased binding of mouse monocytic WEHI 78/24 cells to the aortic vessel isolated from diabetic vs. control mice in all three cohorts (4, 8, and 12 weeks). However, a 3.7% blueberry-supplemented diet suppressed the vascular inflammation in diabetic mice, as shown by a reduced monocyte binding to the aortic vessel at 4, 8, and 12 weeks of treatment (Figure 2A). In addition, we assessed inflammatory markers in the 12-week cohort. The mRNA expression of inflammatory chemokines (MCP1 and IL8) and VCAM1 greatly increased in diabetic vs. control mice (Figure 2B). However, 3.7% blueberry supplementation reduced the expression of these inflammatory molecules in diabetic mice (D12B3 vs. D12) (Figure 2B).

### 3.3. Dietary Blueberries Dose- and Time-Dependently Improve Diabetes-Induced Gut Dysbiosis

**Figure 3:**
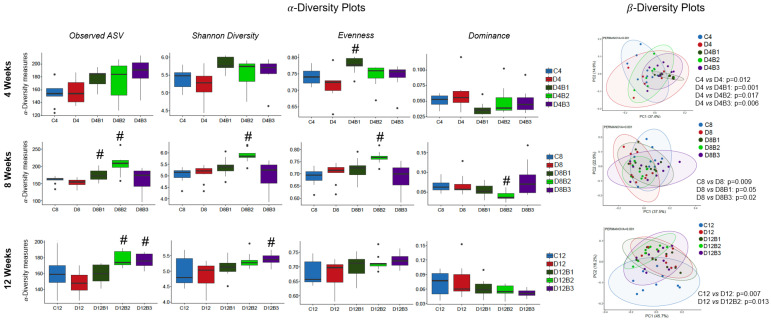
α-Diversity indices and β-diversity of gut microbial communities (4-week, 8-week, and 12-week cohorts). C4, C8, and C12: non-diabetic (*db*/*+*) mice fed a standard diet for 4, 8, or 12 weeks; D4, D8, and D12: diabetic (*db*/*db*) mice fed a standard diet for 4, 8, or 12 weeks; D4B1, D8B1, and D12B1: diabetic (*db*/*db*) mice fed 1.23% freeze-dried blueberry (FD-BB)-supplemented diet for 4, 8, or 12 weeks; D4B2, D8B2, and D12B2: diabetic (*db*/*db*) mice fed a 2.46% FD-BB-supplemented diet for 4, 8, or 12 weeks; and D4B3, D8B3, and D12B3: diabetic (*db*/*db*) mice fed a 3.7% FD-BB-supplemented diet for 4, 8, or 12 weeks. Values are mean ± SEM (*n* = 8–10). * *p* < 0.05, standard diet-fed diabetic mice vs. standard-diet-fed control mice; and # *p* < 0.05, blueberry-fed diabetic mice vs. standard-diet-fed diabetic mice.

**Figure 4:**
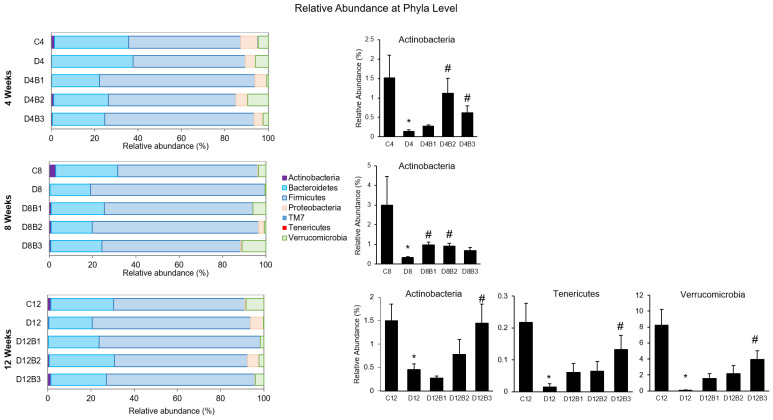
Relative abundance of taxa at phyla level (4-week, 8-week, and 12-week cohorts). C4, C8, and C12: non-diabetic (*db*/*+*) mice fed a standard diet for 4, 8, or 12 weeks; D4, D8, and D12: diabetic (*db*/*db*) mice fed a standard diet for 4, 8, or 12 weeks; D4B1, D8B1, and D12B1: diabetic (*db*/*db*) mice fed 1.23% freeze-dried blueberry (FD-BB)-supplemented diet for 4, 8, or 12 weeks; D4B2, D8B2, and D12B2: diabetic (*db*/*db*) mice fed a 2.46% FD-BB-supplemented diet for 4, 8, or 12 weeks; and D4B3, D8B3, and D12B3: diabetic (*db*/*db*) mice fed a 3.7% FD-BB-supplemented diet for 4, 8, or 12 weeks. Values are mean ± SEM (*n* = 9–10). * *p* < 0.05, standard-diet-fed diabetic mice vs. standard-diet-fed control mice; and # *p* < 0.05, blueberry-fed diabetic mice vs. standard-diet-fed diabetic mice.

**Figure 5:**
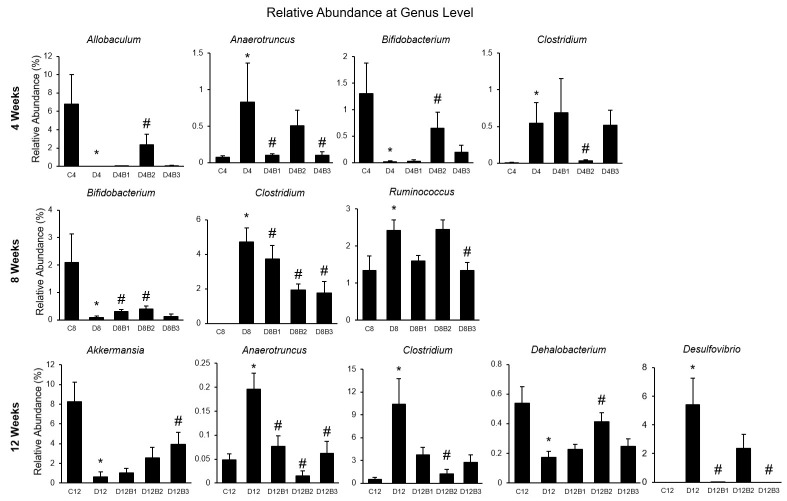
Relative abundance of taxa at genus level (4-week, 8-week, and 12-week cohorts). C4, C8, and C12: non-diabetic (*db*/*+*) mice fed a standard diet for 4, 8, or 12 weeks; D4, D8, and D12: diabetic (*db*/*db*) mice fed a standard diet for 4, 8, or 12 weeks; D4B1, D8B1, and D12B1: diabetic (*db*/*db*) mice fed 1.23% freeze-dried blueberry (FD-BB)-supplemented diet for 4, 8, or 12 weeks; D4B2, D8B2, and D12B2: diabetic (*db*/*db*) mice fed a 2.46% FD-BB supplemented diet for 4, 8, or 12 weeks; and D4B3, D8B3, and D12B3: diabetic (*db*/*db*) mice fed a 3.7% FD-BB-supplemented diet for 4, 8, or 12 weeks. Values are mean ± SEM (*n* = 9–10). * *p* < 0.05, standard-diet-fed diabetic mice vs. standard-diet-fed control mice; and # *p* < 0.05, blueberry-fed diabetic mice vs. standard diet-fed diabetic mice.

α-Diversity indices such as Observed ASV, Shannon Diversity, Evenness, and Dominance (which measure the richness and evenness of the microbial community) were not significantly different in diabetic vs. control mice at the ASV level (Figure 3). However, β-Diversity (which measures the similarity or dissimilarity of the microbial community) was significantly different between diabetic and control mice (Figure 3). Further, blueberry supplementation to diabetic mice significantly altered the β-Diversity that depended on dose and duration of treatment (Figure 3). In the present study, the microbial community was distributed into six major phyla such as Actinobacteria, Bacteroidetes, Firmicutes, Proteobacteria, Tenericutes, TM7, and Verrucomicrobia (Figure 4). Diabetes affected the relative abundance of several phyla and some of them were improved with blueberry treatment (Figure 4). The diabetic mice exhibited a decreased abundance of Actinobacteria in all three cohorts (4, 8, and 12 weeks). Blueberry supplementation increased the abundance of Actinobacteria at 4 weeks (with 2.46% and 3.7% dose), 8 weeks (with 1.23% and 2.46% dose), and 12 weeks (with 3.7% dose) of treatment. In addition, several taxa (at genus level) were altered in diabetes, and most of them were improved with different dosages of blueberry (Figure 5). The relative abundance of the genera such as *Allobaculum* and *Bifidobacterium* was decreased, whereas *Anaerotruncus* and *Clostridium* were increased in diabetic mice in the 4-week cohort. *Bifidobacterium* was decreased, whereas *Clostridium* and *Ruminococcus* were increased in diabetic mice in the 8-week cohort. *Akkermansia* and *Dehalobacterium* were decreased, whereas *Anaerotruncus*, *Clostridium*, and *Desulfovibrio* were increased in diabetic mice in the 12-week cohort. Blueberry supplementation (1.23%, 2.46%, or 3.7% in diet) improved the abundance of these genera that were altered in diabetes at different time points (Figure 5).

### 3.4. Association between Gut Microbial Signature and Vascular Inflammation

**Figure 6:**
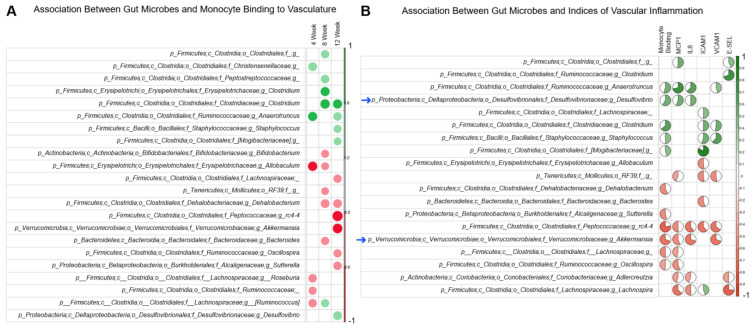
Spearman’s correlation plots: (**A**) Association between gut microbes and monocyte binding to the vasculature (4-week, 8-week, and 12-week cohorts). (**B**) Association between gut microbes and vascular inflammation (12-week cohort).

The time-dependent association between gut microbes (genera) and monocyte binding to vasculature was assessed using the data from 4-week (C4, D4, D4B3), 8-week (C8, D8, D8B3), and 12-week (C12, D12, D12B3) cohorts. Several genera were positively or negatively associated with monocyte binding to the vasculature depending on the duration of the treatment (Figure 6A). We also identified the association between the specific genera and indices of vascular inflammation (monocyte binding to the vasculature and expression of inflammatory molecules such as MCP1, IL8, ICAM1, VCAM1, and E-SEL) in the 12-week cohort using the data from C12, D12, and D12B3 mice. Many of the genera positively associated with the inflammatory markers were also positively associated with monocyte binding to the vasculature (Figure 6B). Similarly, many of the genera negatively associated with the inflammatory markers were also negatively associated with monocyte binding to the vasculature (Figure 6B).

## 4. Discussion

Emerging evidence from epidemiological, clinal, and preclinical studies indicates the vascular beneficial effects of consuming blueberries. However, dietary blueberries’ dose- and time-dependent effects on diabetic vasculature and their association with gut microbes are unknown. In the present study, we determined whether the effect of dietary blueberries on diabetic vasculature is dose/time-dependent and identified whether these effects are associated with specific gut microbes. First, dietary supplementation of blueberries for 4, 8, or 12 weeks suppressed vascular inflammation in diabetic mice at a dosage equivalent to the human consumption of 1.5 cups of fresh blueberries. This effect was not secondary to an improvement in the metabolic milieu, suggesting the direct effect of blueberry bioactives on diabetic vasculature. Second, dietary blueberries ameliorated diabetes-induced gut dysbiosis that depended on the dose and duration of the treatment. Third, opportunistic microbes were positively associated, whereas commensal microbes were negatively associated with indices of vascular inflammation. Finally, dietary blueberries reduced the opportunistic microbe *Desulfovibrio* that was positively associated with vascular inflammation, and increased the commensal microbe *Akkermansia* that was negatively associated with vascular inflammation.

Monocyte binding to the inflammatory vasculature is a key initial event involved in atherosclerosis and is a potential target to prevent vascular disease [3]. Cardiovascular risk factors such as high glucose and dyslipidemia activate the vascular endothelium to secrete inflammatory chemokines (MCP1 and IL8) and adhesion molecules (ICAM1, VCAM1, and E-Selectin), which leads to monocyte binding to the vasculature and the subsequent vascular inflammation [3,13]. In the present study, blueberry supplementation at the nutritional dosage (equivalent to human consumption of ~1.5 cups of fresh blueberries) suppressed monocyte binding to the diabetic vasculature and reduced the mRNA expression of MCP1, IL8, and VCAM1. This is consistent with previous human studies supporting the vascular beneficial effects of dietary blueberries. Blueberry intake was shown to increase endothelium-dependent vessel relaxation in healthy humans in a dose-dependent manner [5]. In the present study, the metabolic parameters that were altered in diabetic mice were not improved with blueberry supplementation. We recently showed that blueberry metabolites suppress endothelium inflammation in endothelial cells isolated from individuals with type 2 diabetes and improve lipotoxicity-induced endothelial dysfunction [4,23]. Our previous and current data suggest that the observed vascular effects of blueberries could be mediated by the direct effect of their circulating metabolites on the vasculature.

Blueberries act as a prebiotic and improve diabetes-induced gut dysbiosis in a dose- and time-dependent manner. A recent study indicated that the effect of flavonoids on the gut microbiome is dosage-dependent, and a significant divergence was observed in the gut microbiome with the habitual intake of high and low flavonoids [24]. Blueberry supplementation improved the relative abundance of several taxa in diabetic mice in all three cohorts. Phyla such as Actinobacteria, Tenericutes, and Verrucomicrobia were decreased in diabetic mice. Actinobacteria is a phylum that includes Gram-positive anaerobic bacteria consisting of numerous commensal genera (such as *Bifidobacterium*, widely used as probiotics) and plays an essential role in gut homeostasis [25,26]. Tenericutes improve cardiovascular risk factors as shown by their association with lower triglycerides and higher HDL levels [27]. Genera belonging to Verrucomicrobia are associated with reduced hypertension in humans [28]. In the present study, blueberry supplementation increased the abundance of Actinobacteria, Tenericutes, and Verrucomicrobia in diabetic mice. Further, commensal genera such as *Bifidobacterium* and *Akkermansia* were decreased, whereas opportunistic genera such as *Desulfovirbio* and *Clostridium* were increased in diabetic mice. *Bifidobacterium* is a popular probiotic, and studies indicate its beneficial effects on cardiovascular disease [29]. *Akkermansia* belongs to the phylum Verrucomicrobia and exhibits anti-inflammatory effects in preclinical models [30,31]. *Akkermansia muciniphila* regulates mucin production in the gastrointestinal tract, host metabolism, and immune response, suggesting it could be a potential target in diseases related to gut dysbiosis [31]. *Akkermansia muciniphila* is also considered a next-generation probiotic, and studies indicate its beneficial effect in lowering hypertension [28]. In the present study, blueberry supplementation increased the abundance of the commensals (*Bifidobacterium* and *Akkermansia*) in diabetic mice, consistent with a previous human study that showed an increase in the abundance of *Bifidobacterium* following blueberry intake [32]. The *Desulfovibrio* genus is a type of sulfate-reducing bacteria responsible for producing cytotoxic hydrogen sulfite [33]. A human study indicated an increased abundance of *Desulfovibrio* in individuals with type 2 diabetes [34]. *Desulfovibrio desulfuricans* aggravates atherosclerosis by increasing intestinal permeability and circulatory lipopolysaccharide in a mouse model of atherosclerosis (ApoE^−/−^ mice) [35]. Gut metabolites produced by *Desulfovibrio* promote inflammatory molecules such as IL6 and IL8 [33]. Taxa belonging to the genera *Clostridium* are Gram-negative obligate anaerobic pathogens strongly associated with inflammation [36]. In the present study, dietary blueberries reduced the abundance of *Desulfovibrio* and *Clostridium*. The role of gut microbes is crucial in metabolizing bioactive compounds of blueberries into circulating phenolic metabolites [4,23]. Therefore, maintaining a healthy gut microbiome is essential to harness the vascular benefits of consuming blueberries. Our current study suggests that the bioactive compounds found in blueberry may serve as prebiotics, promoting the growth of beneficial microbes. This, in turn, could enhance the production of metabolites derived from blueberries and could be one of the possible reasons for the beneficial effects of dietary blueberries observed in the present study.

Spearman’s correlation indicated that several genera are positively or negatively associated with the indices of vascular inflammation. Most importantly, the opportunistic microbes were positively associated with vascular inflammation, whereas commensal microbes were negatively associated with vascular inflammation. The commensal *Akkermansia* is negatively associated with IL8, MCP1, VCAM1, and monocyte binding to the vasculature. The abundance of *Akkermansia* is significantly reduced in diabetic mice, but blueberry supplementation increased the abundance of this genera. Similarly, the opportunistic microbe *Desulfovibrio* is positively associated with IL8, MCP1, and monocyte binding to the vasculature. However, blueberry supplementation suppressed the abundance of *Desulfovibrio*. These data indicate that dietary blueberries could exert beneficial effects on vasculature by suppressing the opportunistic microbes that contribute to vascular inflammation while simultaneously promoting the growth of commensal microbes that help to counteract vascular inflammation.

## 5. Conclusions

Dietary supplementation of blueberries suppressed vascular inflammation and improved gut dysbiosis in diabetic mice that depends on the blueberries’ dosage and treatment duration. Dietary blueberries reduced the opportunistic microbe that was positively associated with vascular inflammation (*Desulfovibrio*), and increased the commensal microbe that was negatively associated with vascular inflammation (*Akkermansia*). Future research is needed to elucidate the role of gut microbes in the production of blueberry metabolites and identify the influence of blueberry-derived microbial metabolites on the vascular effects of blueberries. In conclusion, dietary blueberries may be a potential strategy to improve diabetes-induced vascular complications by modulating gut microbes.

## Figures and Tables

**Table 1:** Experimental design.

Experimental Mice	Diet	Blueberry Dosage (Human Equivalent)	Duration of Treatment		
			4 Weeks	8 Weeks	12 Weeks
*db*/*+* mice (C)	Standard Diet		C4	C8	C12
*db*/*db* mice (D)	Standard Diet		D4	D8	D12
*db*/*db* mice (D)	1.27% Freeze-dried blueberry powder supplemented Diet (B1)	½ cup (80 g)	D4B1	D8B1	D12B1
*db*/*db* mice (D)	2.46% Freeze-dried blueberry powder supplemented Diet (B2)	1 cup (160 g)	D4B2	D8B2	D12B2
*db*/*db* mice (D)	3.7% Freeze-dried blueberry powder supplemented Diet (B3)	1.5 cup (240 g)	D4B3	D8B3	D12B3

C4, C8, and C12: non-diabetic (*db*/*+*) mice fed a standard diet for 4, 8, or 12 weeks; D4, D8, and D12: diabetic (*db*/*db*) mice fed a standard diet for 4, 8 or 12 weeks; D4B1, D8B1, and D12B1: diabetic (*db*/*db*) mice fed 1.23% freeze-dried blueberry (FD-BB)-supplemented diet for 4, 8, or 12 weeks; D4B2, D8B2, and D12B2: diabetic (*db*/*db*) mice fed a 2.46% FD-BB-supplemented diet for 4, 8, or 12 weeks; and D4B3, D8B3, and D12B3: diabetic (*db*/*db*) mice fed a 3.7% FD-BB-supplemented diet for 4, 8 or 12 weeks, respectively.

**Table 2:** Body weight, blood glucose, and body composition in experimental mice.

4 Weeks	C4	D4	D4B1	D4B2	D4B3
Body weight (g)	28.3 ± 0.41	44.5 ± 0.56 *	43.9 ± 0.47	43.6 ± 0.47	44.1 ± 0.20
Blood glucose (Fasting)	53.9 ± 4.37	188.4 ± 13.37 *	186.5 ± 23.26	152.5 ± 10.95	152.3 ± 10.24
Blood glucose (non-fasting)	179.6 ± 8.82	472.1 ± 22.52 *	537.9 ± 23.88	564.4 ± 16.17	568.4 ± 15.13
Body fat (%)	20.5 ± 1.1	68.3 ± 087 *	68.6 ± 0.58	67.3 ± 0.67	67.3 ± 0.51
Lean body mass (%)	67.3 ± 0.59	40.4 ± 0.39 *	40.2 ± 0.34	40.3 ± 0.42	40.4 ± 0.29
Fluid (%)	12.9 ± 0.2	8.3 ± 0.17 *	8.5 ± 0.15	9 ± 0.17	8.9 ± 0.16
8 Weeks	C8	D8	D8B1	D8B2	D8B3
Body weight (g)	28.3 ± 0.41	44.5 ± 0.56 *	43.9 ± 0.47	43.6 ± 0.47	44.1 ± 0.20
Blood glucose (Fasting)	53.9 ± 4.37	188.4 ± 13.37 *	186.5 ± 23.26	152.5 ± 10.95	152.3 ± 10.24
Blood glucose (non-fasting)	179.6 ± 8.82	472.1 ± 22.52 *	537.9 ± 23.88	564.4 ± 16.17	568.4 ± 15.13
Body fat (%)	20.5 ± 1.1	68.3 ± 087 *	68.6 ± 0.58	67.3 ± 0.67	67.3 ± 0.51
Lean body mass (%)	67.3 ± 0.59	40.4 ± 0.39 *	40.2 ± 0.34	40.3 ± 0.42	40.4 ± 0.29
Fluid (%)	12.9 ± 0.2	8.3 ± 0.17 *	8.5 ± 0.15	9 ± 0.17	8.9 ± 0.16
12 Weeks	C12	D12	D12B1	D12B2	D12B3
Body weight (g)	28.3 ± 0.41	44.5 ± 0.56 *	43.9 ± 0.47	43.6 ± 0.47	44.1 ± 0.20
Blood glucose (Fasting)	53.9 ± 4.37	188.4 ± 13.37 *	186.5 ± 23.26	152.5 ± 10.95	152.3 ± 10.24
Blood glucose (non-fasting)	179.6 ± 8.82	472.1 ± 22.52 *	537.9 ± 23.88	564.4 ± 16.17	568.4 ± 15.13
Body fat (%)	20.5 ± 1.1	68.3 ± 087 *	68.6 ± 0.58	67.3 ± 0.67	67.3 ± 0.51
Lean body mass (%)	67.3 ± 0.59	40.4 ± 0.39 *	40.2 ± 0.34	40.3 ± 0.42	40.4 ± 0.29
Fluid (%)	12.9 ± 0.2	8.3 ± 0.17 *	8.5 ± 0.15	9 ± 0.17	8.9 ± 0.16

C4, C8, and C12: non-diabetic (*db/+*) mice fed a standard diet for 4, 8, or 12 weeks; D4, D8, and D12: diabetic (*db/db*) mice fed a standard diet for 4, 8, or 12 weeks; D4B1, D8B1, and D12B1: diabetic (*db/db*) mice fed 1.23% freeze-dried blueberry (FD-BB)-supplemented diet for 4, 8, or 12 weeks; D4B2, D8B2, and D12B2: diabetic (db/db) mice fed a 2.46% FD-BB-supplemented diet for 4, 8, or 12 weeks; and D4B3, D8B3, and D12B3: diabetic (db/db) mice fed a 3.7% FD-BB-supplemented diet for 4, 8, or 12 weeks, respectively. Values are mean ± SEM (*n* = 8–10). * *p* < 0.05, standard-diet-fed diabetic mice vs. standard-diet-fed control mice.

## Data Availability

Raw sequences are available at the NCBI Sequence Read Archive (SRA) under accession no. PRJNA996159.

## Supplementary Materials

The following supporting information can be downloaded at: https://www.mdpi.com/article/10.3390/antiox12081527/s1, Supplementary Figure.
