# Supplementary material for: Dose- and Time-Dependent Effect of Dietary Blueberries on Diabetic Vasculature Is Correlated with Gut Microbial Signature
Source: Antioxidants (Basel). 2023 Jul 30;12(8):1527. doi: 10.3390/antiox12081527 (PMC10451530; doi:10.3390/antiox12081527)

Supplementary Material

Supplementary Figure. Rarefaction plots for gut microbes (4 (4-week, 8-week, and 12-week cohorts). C4, C8, and C12: non-diabetic (*db/+*) mice fed a standard diet for 4, 8 or 12 weeks; D4, D8, and D12: diabetic (*db/db*) mice fed a standard diet for 4, 8 or 12 weeks; D4B1, D8B1, and D12B1: diabetic (*db/db*) mice fed 1.23% freeze-dried blueberries (FD-BB) supplemented diet for 4, 8 or 12 weeks; D4B2, D8B2, and D12B2: diabetic (*db/db*) mice fed a 2.46% FD-BB supplemented diet for 4, 8 or 12 weeks; and D4B3, D8B3, and D12B3: diabetic (*db/db*) mice fed a 3.7% FD-BB supplemented diet for 4, 8 or 12 weeks.

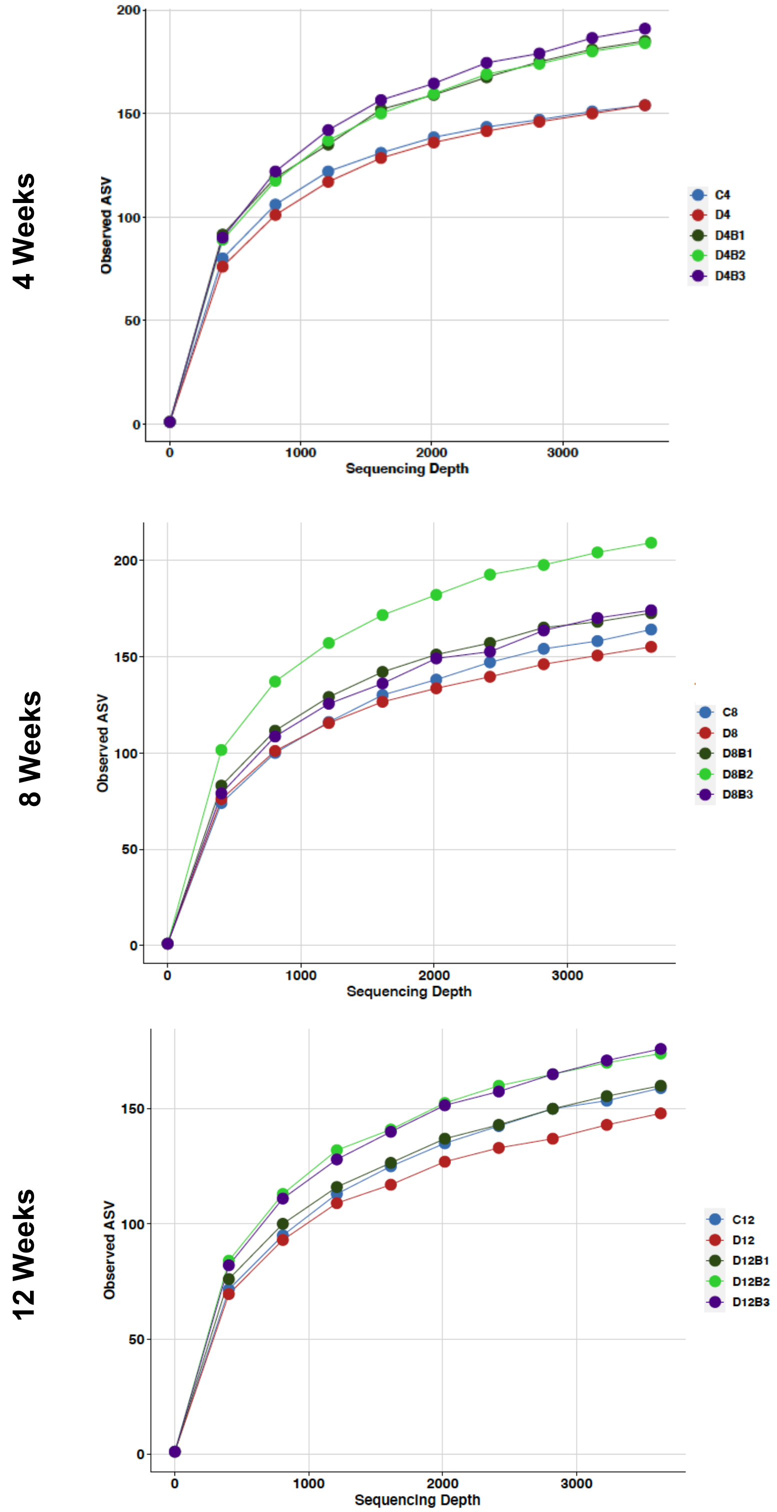

Supplement: Supplementary file 1 [file antioxidants-12-01527-s001.zip › antioxidants-2503414-supplementary.pdf]
